# Understanding the Role of Standards in the Negotiation of a Healthy Built Environment

**DOI:** 10.3390/su12239884

**Published:** 2020-11-26

**Authors:** Rosalie Callway, Helen Pineo, Gemma Moore

**Affiliations:** UCL Institute for Environmental Design and Engineering (IEDE), London WC1H 0NN, UK

**Keywords:** health, wellbeing, standards, built environment, negotiation, urban sustainability, implementation

## Abstract

A growing number of international standards promote Healthy Built Environment (HBE) principles which aim to enhance occupant and user health and wellbeing. Few studies examine the implementation of these standards; whether and how they affect health through changes to built-environment design, construction, and operations. This study reviews a set of sustainability and HBE standards, based on a qualitative analysis of standard documents, standard and socio-technical literature on normalization and negotiation, and interviews with 31 practitioners from four geographical regions. The analysis indicates that standards can impact individual, organizational, and market-scale definitions of an HBE. Some changes to practice are identified, such as procurement and internal layout decisions. There is more limited evidence of changes to dominant, short-term decision-making practices related to cost control and user engagement in operational decisions. HBE standards risk establishing narrow definitions of health and wellbeing focused on building occupants rather than promoting broader, contextually situated, principles of equity, inclusion, and ecosystem functioning crucial for health. There is a need to improve sustainability and HBE standards to take better account of local contexts and promote systems thinking. Further examination of dominant collective negotiation processes is required to identify opportunities to better embed standards within organizational practice.

## 1 Background

The design, layout, and functional features in the built environment can promote or damage the health and wellbeing of occupants and users. Numerous studies report the benefits of healthy buildings, public spaces, and neighbourhoods through reducing exposure to pollutants, increasing physical activity, supporting social connection, and other mechanisms [1–7]. Since the early millennium, standards, and frameworks have proliferated globally which set out design criteria and principles reported to enhance the sustainability of the built environment, including for human health [8–12].

There are over 50 standards, or rating systems, for buildings and neighbourhoods globally (Appendix A). The oldest and most well-known standards, Building Research Establishment Environmental Assessment Method (BREEAM) and Leadership in Energy and Environmental Design (LEED), launched in 1990 and 2000, respectively, include criteria related to environmental sustainability and health [13]. Standards are applicable at different stages (e.g., design to operation), building types (e.g., schools and residential), and scales (e.g., individual buildings and neighbourhoods). The more recent healthy built environment (HBE) standards, such as Fitwel® and the WELL Building Standard (launched in 2014 and 2015, respectively) follow the structure of established standards and their assessment criteria are aligned to BREEAM and LEED to allow certification to multiple systems.

Academic literature about built environment standards has particularly focused on their ‘value-laden’ substantive content regarding the range and balance of criteria or intentions they seek to promote [10–12,14,15]. For example, in sustainability standards there is a reported imbalance towards global environmental intentions over more localised social intentions [14,16]. This imbalance may be linked to a preference for more readily measurable criteria (e.g., calculated energy use) over more qualitative factors (e.g., subjective wellbeing) [16,17]. Although there is limited research on the implementation of HBE standards, there are similar concerns that they may prioritise criteria that are more readily quantifiable, such as rates of asthma or sick building syndrome, as opposed to more qualitative concerns, such as a sense of wellbeing [8]. The concept of a healthy built environment is complex due to the wide range of factors that can influence health (e.g., social, environmental and economic), many of which are not affected solely through changing the design of the built environment.

There is a gap in the literature about how developers, planners, residents, and design teams perceive and apply built environment standards [10,14]. Both the substantive content and functional application of standards are important concerns in understanding their potential impact. As such, there is a need to examine both how people perceive and use standards in practice, and whether they actually contribute to changing the decisions, practice, and material outcomes associated to a project. There is some evidence that built environment standards can provide various benefits, such as improving occupant health, real estate values, and development quality [3,18,19], however, policy-makers can require standards for administrative convenience, without necessarily understanding the distinction between alternative frameworks and how they define and target health and sustainability intentions [16,20]. Developers may not fully comply with voluntary standards and some standards may result in unintended consequences which undermine their health and sustainability intentions [8,21].

 In this paper, we present the key findings of a study examining how standards function to promote healthy development, through a qualitative analysis of different discourses from literature and practitioners’ experiences. Theoretically, we draw upon the implementation of science literature regarding socio-technical processes of normalisation and negotiation to understand the work that standards can do to promote health and wellbeing. The main aim of this study is to better understand whether and how standards influence built environment development processes and outcomes that aim to be ‘healthy’. We seek to unpack the following questions: How is the concept of ‘health’, as defined by HBE standards, used to shape narratives and material responses? How do practitioners perceive HBE standards and what are the drivers for selecting and implementing the standards? Are standards used to improve practice, legitimise existing practice or even act as a barrier to change?

## 2 An Overview of Built Environment Standards

Standards can be understood as tools used by public or private sectors to create ‘agreed-upon rules for the production of (textual or material) objects’ [22]. They create normative frameworks through which to evaluate the quality of a system or object. This paper focuses on a core group of sustainability and HBE standards identified by interview participants. In socio-technical theoretical terms, sustainability and HBE standards are complex ‘actants’ in that they can affect decisions and material actions relating to the built environment by specifying normative design, performance, terminological, and procedural requirements [16,21].

Built environment standards aim to improve the quality of buildings, neighbourhoods, and urban places, with multiple benefits for different actors, including: enhanced organisational reputation; increased real estate value; consolidating regulatory requirements; a smoother planning application process; and adopting upfront evaluative practices to reduce the risk of costly changes later in a development cycle [14]. How standards are perceived and applied can also impede the quality of a development [8,14,21,23]. How terms like ‘sustainability’, ‘health’, and ‘wellbeing’ are defined by standards is important as this can affect how people interpret those concepts in practice when implementing a project.

There has been some research reviewing both the substantive content and implementation of sustainability standards [24,25]. There has been less examination of HBE standards which have come into the market more recently. A review of the literature on sustainability and HBE standards highlights four interconnected themes about how standards can affect built environment projects: negotiation of intentions; systems thinking; contextual relevance; and embedding reflexivity.

### 2.1 Negotiating Intentions—External and Internal Drivers

The design, development, and operation of new buildings and communities are becoming increasingly collaborative processes requiring complex negotiation of a plethora of intentions which sometimes complement, and sometimes conflict, with each other [3]. It is this process of negotiation that needs to be more transparent in order to understand the role that standards play in shaping built environment outcomes. There are a range of drivers, external and internal to an organisation, that can enable or constrain the selection and implementation of standards and how practitioners interpret specific standard intentions to take decisions and material actions. External drivers include legal rules and regulations, normative policies and guidance, and the cultural practices that can set expectations on individuals and organisations regarding how they make decisions, adopt certain perceptions and undertake actions [26,27]. Internal drivers that may influence how an organisation, group, or individual negotiates development intentions include the concept of capability, which refers to the resources available to organisations or individuals (knowledge, skills, access to decision-making, time available, finance) affecting their ability to engage in negotiation processes [28–31]. Second, the temporal mode of agency that different actors adopt when considering different development intentions is highlighted, including: (i) iterative agency, basing intentionality on past behaviours or practices; (ii) practical-evaluative agency, where a short-term pragmatic approach is adopted when negotiating intentions, prioritising immediate concerns; and (iii) projective agency, where longer term intentions perceived as holding future benefits are prioritised [32–34]. Third, the degree of coherence between standards and the sense-making work of individuals and organisations was highlighted, where negotiation is affected by the shared and individual understandings of intentions and of different standards to deliver those intentions [35]. Finally, the concept of reflexivity was raised, regarding how organisations and individuals appraise, formally and informally, the work of a standard. The learning that participants experience through appraisal serving to further shape negotiation of intentions. Such appraisal and learning can affect whether participants engage in static (business as usual), regressive (opposing), or progressive (supporting) responses to standards [24].

Particularly dominant in many developments are the extrinsic and intrinsic drivers associated to financial viability or value management [16,21]. Both public and private sector actors can focus on (comparatively) short-term financial concerns with less emphasis on longer-term built environment intentions, such as enhancing equity and ecosystem services [13,36–39]. Boyko and Cooper (2011) [40] suggest this dominance is due to organisations failing to think holistically about people and places, prioritising the business case over human needs.

### 2.2 Systems Thinking

There is a common critique that standards lack a systems-based approach in their treatment of ‘discrete’ intentions—failing to address interdependencies that can exist [11,41]. This is despite a systemic or holistic approach being a central principle of sustainability, where better health can deliver benefits to poverty reduction, increase equality, stimulate economies, and vice versa [42]. Systems thinking requires consideration of impacts beyond the physical boundaries of a site and over time (i.e., intra and intergenerational equity). Standards that predominantly focus on an individual building or community in isolation, or one specific timeframe, can miss potential synergies between different intentions [43]. For example, by promoting the intentions of ‘security and safety’ a standard might encourage overtly protective designs, such as gated developments, which can actually reduce the sense of wellbeing and security for those living and working around a site [44,45].

The unintended consequences of disconnected standard intentions include the trade-offs resulting from gentrification [16,46]. For example, standards that promote green infrastructure may improve a local green space, but also increase real estate prices and displace people on lower incomes, so-called ‘environmental gentrification’ [24,47].

Thinking in systems also raises questions about boundaries, in other words, for how long a developer can be expected, by a standard, to take account of potential impacts of a project to present and future generations [48,49].

### 2.3 Contextual Relevance

The selection and balance of standard criteria or intentions is a further problematic area. Critics argue that standards remain overly prescriptive, establishing a ‘one-size fits all’ approach, and restricting adaptive, user-led, inclusive, and creative responses which deliver more locally appropriate outcomes [16,21,43,50,51]. Built environment standards typically contain ‘core’ criteria that all applicants are expected to meet and optional criteria that can be selected in response to local context. Standards are not always prescriptive regarding the means by which criteria are met and they can support ‘innovation’ by awarding points for creative design solutions, processes, and technologies [14]. Thus, standards are not always wholly prescriptive, although they do tend to focus more on measurable outputs rather than broader outcomes that might be more open to contextual interpretation.

The calculation of an overall score or rating for a certified building or community often involves normalising values and weighting individual criteria or categories so that some issues are intentionally more important than others [13,52]. However, differentiating weights involves subjective decisions about the relative importance of sustainable design priorities. Weighting in sustainability standards may emphasise actions deemed of global significance, encouraging applicants to deprioritise local priorities or bypass local deliberation about interdependencies and trade-offs [15,53,54].

### 2.4 Embedding Reflexivity

The formal and informal evaluative practices that organisations undertake throughout a project affect how an organisation reflexively learns from, interprets and responds to a standard [21,24]. The requirement to keep track of progress is thought to promote self-regulation and quality assurance during the implementation of standards [28]. To that end, there has been increased adoption of Post Occupancy Evaluation (POE), including RIBA’s Soft Landing approach, Social Returns on Investment and Social Sustainability Impact Assessment [41,55,56]. Reflexivity (or responsiveness) to evaluative information is a concern when the use of a standard produces less positive evidence of project implementation. This should point to a need to examine why decisions which could result in negative outcomes (e.g., poor health) are occurring, identify potential barriers and opportunities for change in site design, practice, and even identify a need to make changes in a standard. A key concern is therefore whether standards help to embed evaluative reflexivity into decision-making processes and practices [14,15,24,57,58]. Furthermore, to what extent can standards assign responsibility to a developer or other actors to monitor and respond to development impacts, and who has sufficient resources and capacity to address those impacts over time?

The interpretation and responses to evaluative practices can change at different project stages, as different actors come to dominate decision-making [24,32]. Evaluation at earlier stages does not necessarily translate into material changes in procurement, construction, and operations later [17,24,58]. Such static or even regressive evaluative responses can be linked to a number of factors, including: a failure to periodically assess activities; risk adversity by more ‘traditional’ contractors, planners or clients; the prioritisation of more immediate concerns (e.g., finance); perceived costs of implementing a standard; and the voluntary nature of standards [43].

### 2.5 Theoretical Analysis of Organisational Processes, Negotiation and Normalisation

A key focus for the research is *how* standards can drive the normalisation of health-promoting built environment design, construction, and operation. Empirical studies have evaluated differences in indoor environmental quality and health in green buildings compared to conventional buildings, with some indication that the former may be healthier in certain contexts and building types [7,19,59,60]. It remains hard however to extract the degree to which implementation of a built environment standard directly promotes changes in decisions and actions which lead to healthier and more sustainable outcomes. Other factors, external to an organisation, could be equally or more significant in shaping decision-making [17,24]. This might include planning conditions or building regulations which a developer must meet in order to obtain a contract, planning consent or remain viable for investors. It is therefore problematic to make direct links between specific outcomes and requirements in any one standard.

Socio-technical process theories offer potentially useful frameworks to help clarify the ‘black box’ interrelationships between human actors and material artifacts such as standards [61,62]. Standards can be understood as technologies or ‘actants’ used by social actors to shape change [63]. In particular, Normalisation Process Theory (NPT), from implementation sciences, considers how technologies can support changes to routine implementation practices [35,64,65]. This perspective aligns with the central aim of built environment standards which seek to create a normalised framework of principles to change how applicants manage projects. Key concepts from NPT that have a bearing on this study include the idea of coherence, which considers whether actors identify the intentions within a technology (in this case a standard) with personal and/or organisational intentions. NPT reviews the collective actions that are undertaken by actors to make changes to practice. It considers the use of reflexive monitoring or appraisal work that organisations undertake to understand how evaluative practices linked to a standard result in reconfiguration or refinement of practice.

The literature on negotiation also offers relevant concepts to examine how intentions are weighed up, consolidated, and applied within a particular context. Negotiation literature describes two processes in how different intentions are managed within decision-making. First, the *prioritisation* of intentions, which relates to the identification and allocation of importance (weight) to intentions by practitioners. Secondly, the *integration* (or consolidation) of those intentions in order to take decisions [48,66,67]. These concepts from NPT and negotiation are adopted in this study to help unpack whether and how actors respond to standard intentions in the context of a particular built environment project.

### 2.6 Conclusions from Literature Review

Built environment standards are thought of as technical actants that seek to stimulate ‘normalisation’ or embedding of particular intentions in individual and organisational understandings, decision-making, narratives, and material responses [21]. There remains conflicting evidence about whether generalised principles, as advocated in standards, can be used to normalise the creation of health and wider sustainability benefits in new development.

Drawing from the literature, the normalisation of HBE standard intentions may be affected by several factors:

- Extrinsic and intrinsic drivers may constrain or enable the uptake of standards and their intentions. Standards required by policy or law may only lead to a minimal adoption of intentions in comparison to when a company chooses to adopt a standard voluntarily in order to become a ‘market leader’.
- Standards can miss the interdependencies across intentions at different scales and timeframes and in terms of the distribution of benefits (or costs).
- Varying prioritisation of global and local contexts within standards may affect how and whether standards support local health impacts.
- Narrowly focused performance-based standards may lead to specific technical responses that are more measurable but could limit organisational innovation and adaptive problem-solving. Broader design-based or terminologically focused standards may be harder to monitor but allow more responsive interpretation and adaptation within different contexts.
- Practitioner responses to standards at each stage of a project will affect whether standards can be more than superficial tick-box exercises with little impact to becoming reflexive frameworks that affect decisions and actions.

## 3 Methods

The research method had two strands that were iterative rather than sequential. We used secondary data and literature to map and review standards documentation and we conducted a literature review across topics, including built environment standards (in sustainability and health fields) and normalisation and negotiation (from implementation science). We undertook a broad comparison of the substantive and functional scope of a core group of sustainability and HBE standards to gain an overview of their content. Our review and comparison activities focused on standards that were most frequently referred to by interview participants, listed in Table 1. The substantive scope of the standards was contrasted against the Towards Healthy Urbanism: Inclusive, Equitable, Sustainable (THRIVES) framework which collates a range of health and wellbeing intentions that can be promoted through BE projects [68,69]. The THRIVES framework offers a holistic framework through which to examine the scope of standards and the health and wellbeing intentions they seek to address. The framework considers health at interconnected scales, identifying intentions that promote global planetary health (e.g., zero carbon emissions) to local health (e.g., indoor air quality).

The second strand of research involved a qualitative analysis of interviews with built environment and public health practitioners to understand their perceptions of HBE standards and the key drivers involved in promoting health through development. We conducted semi structured interviews with 31 professionals, outlined in Table 2. Participants were located in 6 countries (Australia, China, England, The Netherlands, Sweden, and USA). We obtained ethical approval and all participants were informed and consented. The interviews were approximately 1 h long and were audio-recorded and professionally transcribed. One reason for interviewing a relatively small sample of participants in multiple countries was related to the action research project, of which this analysis formed a part, see Pineo and Moore [70]. The larger project involves working with a landowner and urban health charity in London, Guy’s and St Thomas’ Charity, to inform their approach towards promoting healthy development. The charity partners requested an international scope to the research, however there were budget and time limitations that prevented an in-depth analysis in each country.

To be recruited, participants needed to meet 2 criteria: (1) they were either a built environment or public health professional; and (2) they had experience of working on new developments which have integrated health and wellbeing considerations. Participants were recruited through a purposive and snowball sampling approach [71], using professional contacts and LinkedIn. In total, 62 potential participants were invited to take part, and 31 accepted—a response rate of 50%. Within the sampling process we repeatedly assessed the balance of participants across different geographic areas who also had wider international experience. We recognised that a comparative analysis based on geographical area or professional background was not appropriate, but took the view that the interview data were sufficiently rich to draw provisional findings from the broad cross-section of views we were able to obtain [72].

Data coding was applied with NVIVO software (Version 12.6.0, 2019) using a thematic analysis approach, with deductive and inductive coding methods. The codebook used broad categories informed by the research questions, the literature, and conceptual approach. In the first coding phase, the role of standards emerged as an important driver in relation to promoting a healthy built environment. A second round of coding was then undertaken focusing on the dominant codes associated to standards. Deductive concepts were adopted from the literature review, as well as the theoretical concepts from NPT and negotiation literature. These concepts were used to initially code the standards data, before more dominant drivers underlying the use of standards began to inductively emerge.

## 4 Results

The following section outlines first, the findings from the review of the standard documents, and second, an analysis of the dominant themes emerging from the coding of interview data.

### 4.1 Understanding the Scope of Built Environment Standards

All the standards considered in this study recommend a range of intentions and practices reported to promote different aspects of health and wellbeing. Table 3 highlights the functional scope of the standards, where sustainability and HBE standards often cover multiple scales (individual buildings to community-scale development), building types (commercial offices, residential, and mixed-use sites), and phases of development (design, construction to in-use). All three HBE standards include requirements for POE and verification requirements to assess performance once a building or site is in-use. In total, two sustainability standards, Green Star Communities and Living Building Challenge, encourage developers to assess the impact of financial decisions on sustainability. The HBE standards do not address the impact of financial decisions on health, e.g., how procurement choices can affect local environmental quality or local employment opportunities [36].

There is a degree of variation regarding whether the standards require resident or user feedback. In the HBE group, WELL Communities calls for Health Impact Assessments to be carried out with user engagement, and the Fitwel® standards require annual occupant satisfaction surveys regarding perceptions of design, policies, or operations. All the sustainability standards, except the ‘Living Building Challenge’ and ‘Living Community Challenge’, refer to community consultation and other stakeholders in their community-scale standards. Of the building-scale standards, only BREEAM calls for consultation with communities. None of the HBE standards propose a deliberative and inclusive process to identify and prioritise particular standard intentions, nor do they recommend training to help users or occupants engage in the process of implementing a standard (although the standards do refer to occupant training to implement specific standard intentions, e.g., healthy diet).

Appendix B summarises the health and wellbeing intentions contained in the sustainability and HBE standards, highlighting considerable variation between standards. From comparing the standards, some key insights can be drawn out, in terms of their commonalities and differences as outlined below.

#### 4.1.1 Coverage

There are overlaps between sustainability and healthy built environment standards, particularly in relation to indoor environmental quality. Building-scale sustainability standards tend to cover fewer health and wellbeing intentions than community-scale versions and they refer less to the impacts on the exterior environment. BREEAM appears to be the most comprehensive sustainability standard for health and wellbeing intentions.

Only BREEAM Communities addresses intentions regarding security of tenure and access to health care services. BREEAM Communities, Green Star, and Living Community Challenge refer to promoting access to employment opportunities.

The Regenerative Ecological, Social, Economic Targets (RESET) standard is currently the most-narrow in scope. It focuses almost entirely on promoting indoor air quality through reducing pollutants (carbon dioxide, volatile organic compounds, particulate matter), and managing temperature and humidity. RESET is prescriptive on which elements to measure, how to measure them, and technical systems that applicants should use. Finally, a key point of differentiation between coverage health and sustainability standards is that HBE standards are more likely to require POE.

#### 4.1.2 Equity and Inclusion

In terms of equity and inclusion there are limited references to these concepts in the sustainability standards, although BREEAM Communities covers inclusive design. The Living Building and Communities Challenge standards calls for provision of universal access to green spaces and adoption of the ‘JUST’ programme (www.justorganizations.com) which awards credits for organisations that support volunteering in the local community and local sourcing of products. JUST does not raise broader social equity impacts that can occur at different project stages. Regarding questions of equity and inclusion, WELL calls for the adoption of the JUST programme (regarding employment opportunities) or Global Reporting Initiative (GRI) guidelines for construction and real estate sectors. GRI focuses on the terms of employment regarding equal opportunities (e.g., ensuring living wage, gender pay equity). Fitwel® covers equity and inclusion intentions more broadly than other standards, linking them to various built environment issues (e.g., design, transport, work conditions). Apart from RESET, the HBE standards refer to equity and inclusion intentions that address local access to services and provision of shelter.

#### 4.1.3 Scales of Impact: Ecosystem and Planetary Health

Unlike the sustainability standards, none of the HBE standards have criteria that directly address planetary health, and have limited references to ecosystem services. They principally refer to protecting water, air, and food quality rather than protecting the ecosystems that deliver those qualities. WELL Building has ‘biophilic design’ criteria but only in regard to enhancing the aesthetic design quality, rather than utilising ecosystem services to promote healthy places.

#### 4.1.4 Systems Thinking—Connections between Intentions

The creators of WELL (the International WELL Building Institute, IWBI) and sustainability standards (LEED, BRE, Green Star) have undertaken ‘cross-walk and alignment’ processes highlighting where connections between intentions occur (https://standard.wellcertified.com/well-crosswalks). The existence of the cross-walk process suggests that IWBI are encouraging clients to meet wider sustainability intentions through those standards. For example, IWBI refer to ecology being covered in BREEAM, but they do not make this connection with ecology in relation to LEED or Green Star, so not all WELL projects that are dual-certified with a sustainability standard will necessarily address ecological intentions. The cross-walk documents do not refer to the need for participation in the selection or prioritisation of standards’ intentions, nor do they address topics regarding planetary health, communicable diseases, or systems thinking in relation to potential interactions between differing intentions.

The next section reports interview participants’ views about the use of built environment standards in practice.

### 4.2 Perceptions of Sustainability and Healthy Built Environment Standards in Practice

**Figure 1:**
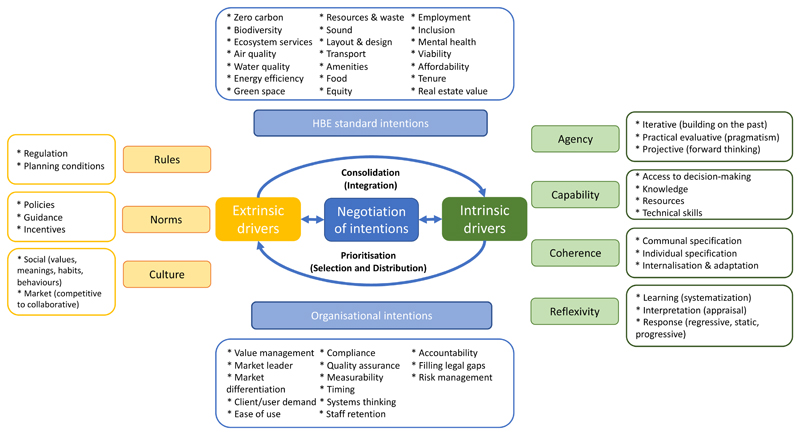
Conceptualisation of the negotiation of HBE standard and organisational intentions in built environment projects.

A central concept emerging from both literature and interviews relates to the **negotiation** of personal and organisational intentions during the delivery of a built environment project and the use of a standard in shaping that negotiation process. Figure 1 brings together these concepts in a single framework, representing the negotiation process that dominant organisational actors (designers, clients, and/or developers) undertake at different stages of BE projects when prioritising (selecting and weighing up) and consolidating (integrating into practice) different intentions.

The negotiation of different intentions and drivers within a built environment project is both a dynamic and interactive process, as different actors come to dominate the process at different stages. The negotiation process and the central concepts are discussed in the following section, focusing on the extrinsic and intrinsic drivers.

#### 4.2.1 Extrinsic Drivers—Rules, Norms and Culture

Extrinsic drivers, such as legislative rules, were often referred to in relation to how standards were selected and applied. Participants from all countries referred to how **normative** planning policies, fiscal incentives, and design guidance affected the prioritisation of specific intentions and standards by design teams or developers. Planning and financial incentives were also used to motivate the adoption of particular standards and intentions. For example, in Seattle, developers that adopted a sustainability standard were allowed to build taller buildings:

“There was a standard that was through our incentive zoning, that you could have additional height if you met LEED Silver” (Planning official, USA).

In comparison, a lack of policy requirements could mean that standards and intentions were passed over:

“We actually looked at BREEAM Communities with them, early on … I don’t think they did it. Again, this was a classic, ‘why should we do it? It’s not in planning. Why do I need to do it?’” (Sustainability consultant, UK).

The effect of **market culture** seemed to play a stronger role in relation to HBE standards. Participants felt that standards needed to demonstrate clear alignment with market intentions:

“Certification [will] always be a very, very limited part of the whole investment. But in order to achieve the investment, what’s the reward? The reward is just the increase in rental [value].” (Sustainability and engineering consultant, China).

Another participant described how the more traditional competitive market culture amongst landlords (as opposed to collaborative) prevented the WELL standard from being integrated into some building operations:

“There were many landlords that just told us, ‘we’re not doing that, we’re not going to monitor and share information, that’s none of your business.’ There’s a lot of that attitude out in the market place … ” (Sustainability consultant, USA).

Noting there were fewer public policies requiring HBE standards, one participant (Sustainability engineer, UK) commented that standards may help to fill policy gaps in key areas, but felt this was a cyclical process, where external requirements grow over time people start to feel there are too many burdens in the market and that people would call for polices and regulations to be “stripped out” again. Then a few years later would people start complaining again about the quality of buildings and call for more regulation. This highlights a push and pull in the competitive market place between the drivers for quality and the drivers for low costs and ease of implementation.

#### 4.2.2 Intrinsic Driver—Temporal Mode of Agency

There was variation in the temporal mode of agency or mindset adopted by designers and developers in relation to negotiation of intentions and whether they were more or less likely to work with a standard. Several participants were critical of clients who they perceived to be overly short-termist (**practical evaluative**) in their mindset. It was perceived that these clients prioritised immediate organisational concerns (including market differentiation, cost control, quality assurance, and ease of implementation), and had limited ambitions of longer-term implications, such as health-related concerns:

“I think it was just like, ‘what’s the fastest, easiest thing we can do?’ because the process has been lengthy” (Sustainability consultant, USA).

The dominance of clients’ expectations affecting the selection and implementation of standards was frequently referred to:

“It’s sometimes difficult to be able to make an actual difference on those projects and I think that, in some part, has got to come from what the client’s expectations are and what they want for the project.” (Sustainability consultant, UK).

Public clients and planners were felt to adopt a longer-term **projective** mindset enabling a broader range of intentions to be considered in contrast to private developers. However, even housing associations, often characterised as having a longer-term outlook, could be pragmatic in their attitudes:

“We pitched [BREEAM Communities] to them a couple of times and it was a classic, ‘well, that looks good, but why should we, as a business …?’—because they were a housing association, also a business, in the housebuilding business, they wanted to make money to build more homes” (Sustainability consultant, UK).

Others indicated however, that as awareness of health concerns and market interest in HBE standards had grown, a more projective or long-term view was beginning to be adopted by some clients and users:

“I think by the nature of who the client is and they want to be an exemplar project and … knowing that there was a growing industry, I guess and awareness of health and well-being, that they wanted that to be a focus as well … but also just the people who would be in that building and I think, knowing that they’ve got a bit more of an awareness of health and well-being” (Sustainability consultant, UK).

Designers and consultants also described adopting a projective approach themselves to encourage clients to select standards. Design teams would proactively reinterpret standard requirements and the narratives around them to demonstrate how standards could align with particular project and organisational intentions:

“We presented to the developer a menu of options and said, ‘well, we can pursue Fitwel certification, we can pursue WELL certification and we walked them through what those meant and how some were more rigorous and more verifiable than others … so that really led them towards creating this sort of combination project where we were not only pursuing LEED certification for the building, but we bundled Fitwel certification into that project as well because it sort of met all of their needs at the price point that they were comfortable with.” (Sustainability consultant, USA).

#### 4.2.3 Intrinsic Driver—Capability

Both design and developer participants sensed that the sector was beginning to acquire knowledge and skills about what a healthy BE meant and how to deliver it, but also recognised that this was somewhat constrained by resources and market perceptions of trendy topics. One designer (UK) described how they were accumulating knowledge through the process of implementing standards, enabling them to demonstrate to developers what could be achieved elsewhere.

Another described their sense of pride at achieving the highest Platinum rating for the WELL standard on one building, describing how this “successful” outcome also encouraged them to enrol further clients to adopt WELL:

“They really tried to achieve the highest rating possible and to do this, they tried to make the healthiest building possible … So if you look up pictures of the building, there is a lot of greenery, fresh air … outer air supply is 50 m^2^ of air per person, so it’s really high standards to achieve a healthy office building and it’s worked out, so it’s nice.” (Engineer, The Netherlands).

Some participants referred to how HBE standards helped promote collaborative sharing of knowledge and joint-working, encouraging people to work across silos and building up a greater knowledge base of what is required to create a healthy development:

“We have a lighting consultant here and we were going through the light criteria in WELL and she’s such a marvel, it was just really engaging to talk to her about, ‘What do you think of this study? What do you think of this research? What do you think about this strategy, does it really work, how much does it work? I think it actually knits together the people that are working with health and well-being and all of these experts because we’re having more profound discussions.” (Architect, Sweden).

Participants remained concerned about the level of knowledge about HBE standards however, in what was perceived as a relatively novel area, as one developer pointed out:

“They’re starting to learn more about WELL Certification, but I haven’t met any architects yet that are knowing more about health and well-being than what I do.” (Developer, Sweden).

There was also a perception, that an overreliance on the evidence and research underpinning HBE standards undermined organisational and individual innovation and knowledge creation:

“I guess they’re relying on what the public health science or the information in WELL that’s saying, ‘it’s better to do it this way’ and it’s hard because how can you question those things, you’re like, ‘okay, somebody has done the research and this is the way to do it” (Sustainability consultant, USA).

It was noted that developers made limited investments in research and innovation, and the time constraints involved in projects could inhibit opportunities to build-up knowledge and experience of implementing a standard:

“It’s a lot more stressful because we have a time limit and are trying to get this knowledge out and working with WELL Certification that is very new for a lot of people, so it gets very frustrating” (Developer, Sweden).

Such constraints on resources meant design teams were more likely to promote the adoption of standard principles without necessarily seeking certification against the standard.

#### 4.2.4 Intrinsic Driver—Coherence

The interviews pointed to a particularly strong alignment between organisational intentions or values and the selection and integration of standards. Better integration of a standard and its intentions by an organisation was linked to a number of organisational intentions, including: the “badge” of quality assurance (urban design consultant, UK) that standards provided; ease of use; boost to a company’s profile; creating market differentiation in the face of competition; and increasing real estate value:

“For some clients, they just want to be seen to be at the front of … the pointy end of the agenda, whatever that agenda is and that, to some extent, makes you look like the good guy in town.” (Sustainability consultant, UK).

The perceived cost and ease with which a standard could be implemented were critical organisational intentions. One sustainability consultant (USA) outlined how WELL requirements for regular checks and ongoing reporting on performance were perceived to be overly onerous by clients and made standard adoption a “difficult sell”. Others highlighted how it was “challenging” for even “progressive” clients to adopt the WELL Standard, as it required significant operational and policy changes at the organisational level. In contrast with those who felt HBE requirements were costly to implement, a couple of participants did feel that HBE standards were easier than sustainability standards to understand however and therefore more likely to be implemented.

If a standard was thought to conflict with organisational intentions, participants would get around this by only selecting those elements they wanted to follow. Some participants would undertake the “bare minimum” requirements (Sustainability consultant, UK) and others would avoid a standard entirely:

“Unless there is some marketable value in you being able to say you are WELL certified, we don’t see much point in you doing it, but we can still help you deliver the principles of it.” (Sustainability consultant, UK).

One participant described how they had avoided a standard not perceived to align with their clients’ intentions, despite the standard being required by the planning authority. They engaged in dialogue with the authorities who agreed to reinterpret requirements rather than requiring implementation of the standard.

#### 4.2.5 Intrinsic Driver—Reflexivity (Learning, Interpretation, and Response)

A number of participants indicated that HBE standards had influenced narrative changes how a ‘healthy’ development is defined. This definition was principally linked to a limited number of more measurable health intentions, such as diet, hygiene, air quality, and thermal comfort. Limited references were made to other issues associated to wellbeing, like fuel poverty and affordability. Some health intentions were entirely missing in participant description of standards, notably regarding: equity and inclusion; ecosystem and planetary health; and systems-thinking.

Few participants considered how HBE standards might affect equity or inclusion, such as in relation to: learning from user experience; promoting coproduction in defining a healthy development; promoting access to education or employment; or enhancing mental health. Participants particularly focused on interior material changes in response to HBE standards, with limited reference to impacts on exterior surroundings, such as access to green spaces and nature. This may, in part be linked to the fact that community-scale standards, such as those by Fitwel® and WELL, are still relatively new and were not discussed by participants in terms of their experiences applying the standards. There was no reference to whether HBE standards encouraged actions relating to ecosystem functioning or planetary health. Such intentions were referred to in relation to the One Planet framework and sustainability standards, where participants felt these frameworks also promoted actions likely to support these wider health benefits. Some participants recognised that a narrow interpretation of health within HBE standards distracted attention from the systems-based mindset that sustainability standards sought to promote:

“So now we have WELL and we have Fitwel and we have all of the very specific, human health standards and rating systems and tools in the market that I think are splitting focus and drawing money and time and energy away from a really big foundation that we’ve been building [through sustainability standards] for a long time” (Sustainability consultant, USA).

There were references to standards altering specific site operations, such as sourcing of soap dispensers and healthy food, and specifications for canteens, gyms, and mediation rooms. Some felt, however, that HBE standards did not lead to more significant changes to organisational-scale practices. Rather, standards were simply designed to fit with ‘business as usual’ practices (sustainability consultant, USA), with clients preferring to do “the same thing over again” (Planning consultant, Australia). Various participants described how the responses to HBE standards could also incur unforeseen conflicts with pre-existing sustainability standards.

“It seemed, at first, like we had won all these battles, with the sustainability conversation for 20 years, that we were finally saving energy and saving water and then, if you read WELL, it looks like they’re asking you to up the energy [consumption] for air and up the energy for light” (Designer, USA) “they had to reorient their cleaning and maintenance policies of things just like the way they order soap, the way it’s dispensed which, again, there is science to back that having it in a cartridge is better than any other way, but then we also found that there was a waste element associated with that, so there were definitely some competing trade-offs and priorities” (Sustainability consultant, USA).

## 5 Discussion

### 5.1 Key Findings

This study suggests that standards are selected and used in a variety of ways, affected by the multiple drivers and intentions that individuals and organisations have to negotiate throughout a built environment project. According to the interview participants, the selection and negotiation of HBE standards and their objectives is linked to a range of emergent concepts, including:

● extrinsic drivers:
  ○ the external requirements in normative policies and dominant competitive market culture;
● intrinsic drivers:
  ○ the short-term pragmatic verses projective visionary modes of agency adopted;
  ○ the capability to understand and invest in a standard;
  ○ the alignment of standard intentions with organisational intentions;
  ○ the evaluative interpretation, learning and responses associated to standard requirements.

This study demonstrates that standards need to better address the processes of negotiation that take place at different times and places in a project cycle. There is a need to encourage applicants to move on from principally using standards to legitimise existing practice, towards seeing standards as strategic tools that should promote internal reflection and responses to HBE and wider sustainability objectives.

Promoting health through standards requires building-in greater lines of accountability, including through more participatory and transparent evaluative approaches. This includes overtly addressing the potential links and conflicts between the differing intentions of different actors. There is a need to actively seek a rebalance of the negotiation of these intentions in relation to existing dominant drivers, intentions, and decision-making processes, particularly regarding procurement, value engineering, traditional construction, and operational practices.

The relatively narrow focus of HBE standards and the dynamic nature of how they are responded to means that standards may have a more limited health impact compared to what could be achieved through more holistic and contextually adaptable healthy development principles. The study finds that sustainability and HBE standards can impact the built environment at various scales of practice (micro, meso and macro):

- Microscale changes in operational practice were referred to, such as regarding room arrangement, selection of sinks and food procurement.
- Mesoscale organisational changes were felt to be more challenging, where clients seemed less willing to pay the additional costs of undertaking more strategic and longer-term changes, such as investing in research and development or engaging occupants in operational changes.
- At a macroscale, standards did seem to affect understandings and narratives about health in the built environment.

### 5.2 Towards Further Embedding of Health and Wellbeing in Built Environment Work

The ‘work’ that standards do [21] and how ‘embedded’ they become in organisational practice [24,73] is thought to be affected by regulations and normative policy requirements, but the role of market culture was more frequently referred to by participants. Participants described how market culture has a strong influence over a client’s mind-set and perception of whether a standard aligns with their organisational intentions, such as perceived costs of implementing standards, and whether standards were seen to contribute to competitive advantage [73–75]. The key concern is whether there is a perceived market demand, and therefore willingness to pay, for built environment changes to improve health by standard applicants (e.g., developers, landlords, and employers) as well as demand from users and occupants. Whilst investing in building features and operations that promote health can be seen as a means for developers to improve competitiveness, perceptions of such commercial benefits can change. This makes standards vulnerable to market perceptions of what is seen as a hot topic or fashionable agenda at any one time. In an analysis linked to this study, Pineo and Moore [70] find that creating a ‘business case’ is an essential part of convincing standard applicants to adopt healthy design measures, including and beyond HBE standards. The case may be built on perceived or measurable benefits (including economic, reputational, and others) that will vary depending on the type of developer, local regulations and other factors.

In agreement with Filzmoser et al. (2016), the likelihood of standards being selected and implemented was also greater when they were perceived to align or cohere with a dominant actors’ sense of identity. As such built environment projects are shaped less by the substantive health intentions (or criteria) of a standard and more by the intentionality and identification of those who dominate decision-making at any one time. For example, designers and architects would describe themselves as enablers in promoting a particular standard or health-related intention. Similarly, early adoption of a new standard might align with a clients’ view of themselves as market leaders and innovators. However, if a standard is principally adopted to meet the intention of self-identification or ‘cognitive legitimisation’ for its own sake, there is a risk that the detailed substantive intentions within a standard will only be weakly embedded into more mesoscale organisational practices [17,28].

Linked to the concept of reflexive learning, Sheeran and Webb (2016) and Schweber (2014) suggest prior experience of standard implementation may initially help to stabilise the link between substantive intentions and organisational practice, where risk-averse clients are reassured by the proven expertise that design teams can bring to that process. This positive effect may weaken over time however, as standard implementation becomes more familiar, automatic, or even taken for granted. This study shows that formal certification was not always viewed as essential, which could mean POE verification never occurs and increases a likelihood of a performance gap. Although Sheeran and Webb (2016) focus on understanding the ties between intentionality and responses at the level of individual behaviour, they make recommendations of potential relevance to organisations. In particular, they advise the formal identification of potential opportunities and obstacles to delivery and then making plans outlining what actions to undertake should obstacles arise. Suitable plans should address: (i) initial preparations (e.g., training, stakeholder engagement, agreement of plans, assignment of roles and responsibilities to address obstacles); (ii) keeping on track (e.g., addressing competing intentions and obstacles); (iii) avoiding escalation of commitment if things are not working well; and (iv) avoiding early withdrawal before new practices are given a chance to establish. This suggests that standards would benefit from a proactive approach to ‘self-regulation’, through dialogue, learning, training, as well as monitoring and reporting activities at specified delivery stages in order to engage, refresh, and reengage actors.

HBE standards do appear to have influenced normative understandings of health and wellbeing for the participants in this study. However, the first wave of HBE standards indicate fairly narrow interpretations of health, restricting how actors conceptualise and potentially deliver projects. This aligns with concerns raised in the wider standards literature about limited definitions of sustainability [21,58]. Additional health intentions could be better promoted by HBE standards through less prescriptive requirements and incorporation of broader principles, including promoting: equity, inclusion and contextual relevance; ecosystems and planetary health; and systems-thinking.

HBE standards do not appear to encourage coproduced definitions of healthy places, reducing the opportunity for projects to address the distinct health and wellbeing inequities experienced by different population groups in specific contexts. Lack of contextual relevance also risks undermining the sense of identification and delivery of long-term sustainability [50]. Which actors are involved in negotiation processes can change at different project stages, affecting how, why, and when different intentions are identified and internalised, and potentially affecting who else is engaged in the process [29,69,74]. Actors can perceive standards (and their purpose) differently at various stages, and therefore it is unsurprising that there is a plurality of responses and narratives around standards. A central concern arises therefore, regarding who is (and is not) engaged in a project, how, and when (linked to questions of actor capability and agency to access and influence negotiations) [29,69], as this will give clear pointers to why different standards and intentions are prioritised or deprioritised at any one time. For example, a failure to adequately engage occupants early in considering both standard selection and implementation is more likely to face obstacles at POE stages when the perspectives of users are likely to be more dominant. Clarifying the scope and methods applied during key negotiation processes is critical to establish dialogue, identify common ground [25,74–76], as well as allow sufficient space to acknowledge that agreement may not always possible [74]. This points to the vital importance of allowing sufficient transparency, time and space for different actors to engage in the negotiation of different project intentions and support ‘just’ decision-making [50,66,77]. Standards could also actively promote investment in ‘cultural competencies’, as outlined by Agyeman and Erickson (2012), where designers, developers, and landlords invest in enabling greater interculturalism and inclusivity. Again, this includes encouraging opportunities for learning, training, and dialogue, so that actors can better reflect upon and respond to differing intentions.

As outlined by Pineo [68], the recent COVID-19 outbreak has further highlighted the importance of addressing planetary health, where studies report how rapid unplanned urbanisation (amongst other factors, such as intensive farming) has been associated with increased pressure on ecosystems and wildlife, raising zoonotic and anthropocentric disease risk [78]. This underscores the importance of promoting ecological enhancement through built environment projects to reduce long-term health risks associated with ecological decline. HBE standards currently do little to address such large-scale lasting health impacts. Although their focus on enhancing interior spatial layout and ventilation would help reduce airborne and contact transmission [79,80]. Similarly, the emphasis on increasing inclusive access to good quality green spaces and natural daylight will be valuable in promoting both physical and mental health [81,82], particularly for those subject to quarantine or lock-downs.

From a systems perspective, participant descriptions of the narrow scope of HBE standards suggest that a holistic approach is lacking, even accounting for the cross-walk process seeking to link sustainability and HBE standards. If a more balanced range of health and sustainability intentions are to be brought into built environment narratives and responses, they will need to be raised in dominant negotiating spaces, including regarding financial priorities and operational decision-making. Health intentions either need to be placed on a comparable scale, e.g., in terms of social returns on investment and natural capital, or through some other mode of evaluative comparison [83]. It would be valuable to review alternative, potentially more integrated, evaluative frameworks that seek to directly affect dominant decision-making negotiations, such as Igloo’s Footprint® (http://www.iglooregeneration.co.uk/footprint/), which seeks to ensure investment decisions are evaluated in relation to core intentions and standards at each stage of a development.

### 5.3 Contribution to Future Research

This study provides an early insight into the implementation of HBE standards in development projects. The research highlights the complexity of collective negotiations in relation to normalising standard principles in built environment projects. Such collective negotiations involve dynamic exchanges that can support or confound the prioritisation and internalisation of intentions that produce narrative and material responses. The study highlights a need to enhance the selection and achievement of health and wellbeing intentions through HBE standards, and support more holistic, inclusive, context-sensitive, and reflexive approaches [16,24,41]. Further examination is required of dominant negotiation processes, e.g., financial decision-making during procurement and cost control processes, to better understand how HBE standards can play a greater role in promoting health and wellbeing within the built environment.

The NPT and negotiation literature have provided a useful conceptual basis through which to examine the role of HBE standards as actants in shaping the design, construction, and operation of the built environment. The need for coherence or identification between intrinsic organisational intentions and the explicit substantive intentions of HBE standards was particularly highlighted by the empirical interviews. The ‘collective action’ or negotiation work that individuals and organisations do in the selection and implementation of standards was especially important in that regard, where actors actively sought, in cooperation with others, to enable or constrain that sense of coherence [35,65,84].

The interviews were initially undertaken with broad research objectives about promoting health in the built environment that did not solely focus on standards. As such, further research could be undertaken to obtain a deeper understanding of the selection and implementation of standards during built environment projects to map out and review real world practice in more detail, particularly in relation to making standards more resilient to political and market changes. It would also be valuable to further examine the interrelationships between sustainability and HBE standards to clarify how the cross-walk process could better contribute towards a more holistic understanding of HBE intentions. The more recent roll-out of the community-scale HBE standards also requires further investigation.

Finally, it would be useful to examine how more flexible frameworks, such as Igloo’s Footprint and the Building with Nature certification, are being interpreted and applied to examine whether they are too generic to drive real changes in practice or if a less prescriptive approach stimulates more inclusive and contextually relevant responses.

## 6 Conclusions

Built environment projects involve complex and dynamic negotiations of multiple organisational and substantive intentions. These negotiations are affected by who is engaged in the process at any one time, as well as by a number of drivers, both internal and external to organisations. Whether and how built environment standards are able to affect such negotiations is also constrained and enabled by these collective negotiation processes.

Although this study only provided an early insight into the implementation of HBE standards, the interviews with participants from different countries suggested that standards do serve to shape narratives about how a healthy built environment is defined and whether standards are adopted. There was also recognition of some material changes in response to standards, where standards align with a competitive market culture and organisational intentions. The risk of standards shaping narratives and material practices related to what is missing in their substantive scope, as well as to these intrinsic and external constraints. Early phase HBE standards have focused on the building-scale and local health impacts, missing critical interactions, as well as ecosystem and planetary health scales, scales that even the cross-walk process with the sustainability standards appear to have missed to date.

HBE and sustainability standards need to promote greater opportunities for inclusive and integrated negotiation processes. This change includes encouraging standard applicants to allow for the greater involvement of different actors in the selection and implementation of standards during a particular project, including those who are more marginalised and less able to engage, as well as enabling broad contextual, larger-scale, and longer-term health intentions to be better reflected in the process.

## Supplementary Material

Appendix

## Figures and Tables

**Table 1:** Built environment standards raised by participants grouped by their primary focus (sustainability or health).

Sustainability standards	Building Research Establishment Environmental Assessment
	Method (BREEAM) New Construction and BREEAM Communities (Building Research Establishment, Watford, UK)
	Leadership in Energy and Environmental Design (LEED) New construction and Neighbourhoods (Green Building Council, Washington, DC, USA)
	Green Star Buildings and Communities (The Australian Green Building Council, Sydney, Australia)
	Living Building and Living Communities Challenges (International Living Building Institute, Seattle, WA, USA)

Health standards	WELL Building and WELL Communities (International Well Building Institute, New York, NY, USA)
	Fitwel^®^ (Centre for Active Design, New York, NY, USA)
	"Regenerative Ecological, Social, and Economic Targets" (RESET), (GIGA, Shanghai, China)

**Table 2:** Participant professions.

Profession	No. of Participants
Architecture	5
(Sustainability and) Engineering	4
Planning and urban design	9
Project management	2
Property development	1
Public Health	4
Research (Housing association)	1
Sustainability	5

**Table 3:** Functional scope of sustainability and healthy built environment standards.

	Sustainability Standards				Healthy Built Environment (HBE) Standards		
Functionality	BREEAM	LEED	Green Star	Living Building	WELL	Fitwel®	RESET
Building and community scale	•	•	•	•	•	•	•
Mix of use: residential—commercial	•	•	•	•	•	•	• Interior only
Multi-stage developments	•	•	•	•	•	•	No—mainly operations
POE monitoring	Some standards and criteria	Not mandatory	Within 24 months of design review	After 12 months	Occupant surveys and data reporting	Occupant surveys	Continuous monitoring and reporting
User consultation	For mandatory criteria	Community standard	Community standard	No	Community standard (HIA)	•	No
Procurement requirements	Innovation credit	No	Innovation credit	Proximity of sourcing	No	No	No
Financial reporting	No	No	Innovation credit	•	No	No	No
Third party verification	• On or offsite	• Onsite	•	•	• Onsite	• Off site	• Air quality monitoring systems
Standard type	Design, terminological and performance	Design, terminological and performance	Design, terminological and performance	Design, terminological and performance	Design, terminological and performance	Design, terminological and performance	Performance

Key = • indicates that a feature or requirement is included in the standard. Websites for each standard are listed in Appendix C.
