# Supplementary material for: Understanding the Role of Standards in the Negotiation of a Healthy Built Environment
Source: Sustainability. Author manuscript; Available in PMC 2021 Jan 5. (PMC7116544; doi:10.3390/su12239884)
Supplement: Appendix [file EMS107538-supplement-Appendix.pdf]

Appendix A

Table A1. Sustainable and Healthy Built Environment Standards Internationally.

Country	Sustainable/Healthy Built Environment Standard
Australia	Green Star, National Australian Built Environment Rating System (NABERS)
Brazil	GBC Brasil CASA
Canada	Green Key, Built Green, Green Globes, RESET
China	3 Star, Assessment Standard for Healthy Building (T/ASC02-2016)
Colombia	Casa (Colombia)
Egypt	TARSHEED
France	HQE
Germany	DGNB System
Hong Kong SAR, China	BEAM Plus
India	Indian Green Building Council (IGBC) Rating System
Indonesia	Greenship
Ireland	Home Performance Index
Italy	GBC Home, GBC Historic Building, GBC Quartieri, GBC Condomini
Japan	CASBEE, DBJ Green Building Certification
Kazakhstan	OMIR
Kenya	Green Star SA Kenya
Korea	Korea Green Building Certification
Latvia	BREEAM-LV
Lebanon	ARZ rating system
Malaysia	Green Building Index
Mexico	LEED Mexico
New Zealand	Homestar, NABERSNZ
Norway	BREEAM-NOR
Pakistan	Sustainability in Energy and Environmental Development (SEED)
Philippine	BERDE
Singapore	BCA Green Mark
South Africa	Green Star SA
Spain	VERDE
Sri Lanka	GreenSL
Switzerland	Swiss DGNB System
Taiwan	Green Building Evaluation System (EEWH)
Turkey	CEDBIK-Konut Green building certification system
United Kingdom	BREEAM, Code for Sustainable Homes, Home Quality Mark, Livewell, One Planet Living, Building with Nature
United Arab Emirates	PEARL (Abu Dhabi)
United States	EDGE, Investor Confidence Project (ICP), Enterprise Green Communities, Energy Star, Fitwel®, LEED, Living Building / communities challenge, Net Zero Energy Building (NZEB), WELL Building Standard, Zero Waste
Vietnam	LOTUS

Appendix B

Table A2. Substantive Scope of Built Environment Standards in Relation to Health-Related Topics in the THRIVES Framework.

		Sustainability Standards								Health Standards				
**Scale**	**Health Topic**	**BREEAM NC**	**B C**	**LEED NC**	**LEED N**	**Green Star D&B**	**GSC**	**Living Building C**	**LCC**	**WELL**	**WELL C**	**Fitwel MT**	**Fitwel C**	**Reset**
**Planetary health**	Biodiversity	•	•		•	•	•	•	•					
	Zero carbo	•	•	•		•	•	•	•					
	Natural resources	•	•	•	•	•	•	•	•					
**Regional ecosystem services**	Water	•	•	•	•	•	•	•	•	•	•	•	•	
	Air	•	•	•	•	•		•	•	•	•	•	•	•
	Climate regulation	•	•				•	•	•					
	Food	•	•				•	•	•	•	•	•	•	
	Soil	•	•		•			•	•	• Lead pollution				
	Waste and sanitation	•	•	•	•	•	•	•	•	•	•			
	Energy	•	•	•	•	•	•	•	•	•				
	Other products (e.g., timber)	•	•	•	•	•	•	•	•	•				
	Culture and recreation	•	•		•		•	•	•		•	•	•	
	Mobility	•	•	•	•	•	•	•	•	•	•	•	•	
**Neighbourhood**	Retail and food	•	•		•		•	•	•	•	•	•	•	
	Green (blue) infrastructure	•	•	•	•			•	•		•	•	•	
	Employment		•				•	•	•					•
	Health care	•	•								•	•	•	
	Active transport	•	•	•	•	•	•	•	•	•	•	•	•	
	Education/skills	•	•		•		•	•	•	•	•	•	•	
	Inclusion/equity		• Inclusive design					• Work	• Work	• Work	• Work	•	•	• Reporting
	Decision-making	• Some issues	• Some issues		•		•				• Health Impact Assessment	•	•	

Table A2. *Cont.*

**Building**	Thermal comfort	•	•	•		•				•	•	•		
	Acoustic comfort	•	•	•		•			•	•	•		•	
	Lighting (internal)	•	•	•	•	•		•		•	•	•		
	Lighting (external)	•	•	•	•	•		•	•		•	•	•	
	Indoor Space		•						•		•			
	Visual comfort	•	•	•		•		•		•	•	•		
	Indoor air quality	•		•		•		•		•	•	•		•
	Affordability		•		•	• Innovation	•				•			
	Tenure		•										• Housing mix	
	Local env. Quality	•	•	•				•	•		•	• Air pollution		
	Security and safety	•	•			•	•		•		•	•	•	
	TOTAL	28	32	17	19	18	19	24	24	17	24	18	17	4

Key: BREEAM NC = New Construction (building-scale); BC = BREEAM Communities; LN: LEED New construction (Building scale); LN: LEED neighbourhoods; Green Star D and B = Design and Build (building-scale) GSC = Green Star Communities; Living building challenge (building scale); LC = Living Communities Challenge; WELL (building-scale); WELL C = communities; Fitwel MT = Multi-Tennant (building-scale); Fitwel C = Fitwel communities. • = presence of a standard requirement relating to that health topic.

Appendix C

Standard Website Links

- BREEAM New Construction 2018 (accessed on 01 June 2020): https://www.breeam.com/NC2018/
- BREEAM Communities (2012) (accessed on 01 June 2020): https://www.breeam.com/communitiesmanual/
- LEED standards (accessed on 01 June 2020): https://www.usgbc.org/leed
- Green Star standards (accessed on 01 June 2020): https://new.gbca.org.au/rate/rating-system/
- Living Building Challenge (accessed on 01 June 2020): https://living-future.org/lbc/
- Living Communities Challenge (accessed on 01 June 2020): https://living-future.org/lcc/
- WELL Building Standard (accessed on 01 June 2020): https://standard.wellcertified.com/well
- WELL Communities Standard (accessed on 01 June 2020): https://www.wellcertified.com/certification/community
- FITWEL (accessed on 01 June 2020): https://www.fitwel.org/resources/#v2-1
- RESET (accessed on 01 June 2020): https://reset.build/standard

Note: the table indicates whether a standard refers to requirements regarding a specific topic (• indicating a reference, or blank if no reference) as criteria for projects to address either during design, construction or operation. The methods involved conducting a word search in standards documents for terminology related to themes in the THRIVES Framework. For example, for standard criteria relating to 'Biodiversity' words referring to ecology, wildlife, nature, and biological diversity were also searched for. Similarly, regarding 'zero carbon', words referring to carbon neutral were also searched. Where a topic is only referred to in a limited or specific way then an additional note is made, e.g., Fitwel® only refers to local environmental quality in relation to 'air quality' and does not refer to soil, noise, water quality.
